# Stifled Motivation, Systemic Neglect: A Cross-Sectional Analysis of Inactivity in Post-Chemotherapy Cancer Survivors in the Middle East and North Africa Region

**DOI:** 10.3390/cancers17203375

**Published:** 2025-10-19

**Authors:** Mariem Gaddour, Maha Ammar, Leila Ben Fatma, Halil İbrahim Ceylan, Ines Loubiri, Nedra El Feni, Sonia Jemni, Luca Puce, Nicola Luigi Bragazzi, Ismail Dergaa

**Affiliations:** 1 Physical Medicine and Rehabilitation Department, Sahloul University Hospital, Faculty of Medicine of Sousse, University of Sousse, Sousse 4002, Tunisia; gaddour.mariem@famso.u-sousse.tn (M.G.); ammar.maha.1995@gmail.com (M.A.); loubiriiness@famso.u-sousse.tn (I.L.); sonia.jemni@famso.u-sousse.tn (S.J.); 2 Oncology Department, Farhat Hached University Hospital, Faculty of Medicine of Sousse, University of Sousse, Sousse 4002, Tunisia; leila.benfatmasassi@famso.u-sousse.tn; 3 Physical Education and Sports Teaching Department, Faculty of Sports Sciences, Atatürk University, Erzurum 25240, Turkey; 4 Physical Medicine and Rehabilitation Department, Ibn Jazzar University Hospital, Kairouan 3100, Tunisia; nadra.elfani@famso.u-sousse.tn; 5 Department of Neuroscience, Rehabilitation, Ophthalmology, Genetics, Maternal and Child Health (DINOGMI), University of Genoa, 16132 Genoa, Italy; luca.puce@medicina.unige.it; 6 Laboratory for Industrial and Applied Mathematics (LIAM), Department of Mathematics and Statistics, York University, Toronto, ON M3J 1P3, Canada; 7 High Institute of Sport and Physical Education of Ksar Said, University of Manouba, Manouba 2010, Tunisia; ismail.dergaa@issepks.u-manouba.tn

**Keywords:** cancer fatigue, exercise, health behavior, health policy, patient activation, quality of life, sedentary behavior, socioeconomic factors, survivorship, Tunisia

## Abstract

**Simple Summary:**

Physical activity is a cornerstone of better survival and quality of life after cancer, yet participation remains low, especially in the Middle East and North Africa (MENA) region. This Tunisian study, the first of its kind, examined barriers and facilitators to activity among 120 survivors three months after chemotherapy. Using validated questionnaires, we found a dramatic, sharp decline in exercise: moderate activity dropped from 31.1% before treatment to only 1.7% after. Fatigue was universal, with 21% reporting severe fatigue, and cognitive barriers such as low motivation and negative beliefs were most common. Patient activation also played a key role, as less engaged individuals faced more barriers. Worryingly, only a quarter of survivors received guidance on exercise from healthcare professionals. Statistical analyses showed that severe fatigue, low activation, and advanced cancer stage were independent predictors of inactivity. The Physical Activity Barriers After Cancer scale proved reliable in identifying those at risk. These findings highlight a multidimensional challenge: survivors face both physical and psychological obstacles, compounded by weak healthcare support. The study calls for adapted, multidisciplinary interventions to reduce fatigue, address cognitive barriers, and better engage healthcare providers in exercise promotion across Tunisia and the wider MENA region.

**Abstract:**

**Background:** Physical activity provides substantial survival and quality-of-life benefits for cancer survivors, yet participation remains suboptimal globally, particularly in the Middle East and North Africa (MENA) regions. This study represents the first comprehensive examination of physical activity barriers and facilitators among Tunisian cancer survivors. **Methods:** This cross-sectional study recruited 120 cancer survivors ≥3 months post-chemotherapy completion from University Hospital Farhat Hached, Sousse, Tunisia (October–December 2024). Participants completed validated questionnaires via structured telephone interviews: the International Physical Activity Questionnaire Short Form (IPAQ-SF), the Physical Activity Barriers After Cancer scale (PABAC), the Fatigue Assessment Scale (FAS), and the Patient Activation Measure (PAM-13). Statistical analyses included descriptive statistics, receiver operating characteristic (ROC) analysis, correlation analyses, and multivariable regression modeling with Bonferroni correction for multiple comparisons. **Results:** Participants (mean age 51.89 ± 10.2 years, 73.9% female) demonstrated significant physical activity declines post-chemotherapy: moderate activity decreased from 31.1% to 1.7% (*p* < 0.001), median intensity declined from 297 to 44 MET-min/week (*p* < 0.001). Mean PABAC score was 29.72 ± 5.13, with cognitive barriers predominating (2.85 ± 0.58). Fatigue was universal (100%), with 21% reporting severe fatigue (FAS ≥ 35). Only 26.1% received exercise guidance from healthcare professionals. PABAC demonstrated excellent predictive performance for physical inactivity (AUC = 0.805, 95%CI: 0.724–0.887). Independent predictors of higher barriers included fatigue severity (β = 0.466, *p* < 0.001), low patient activation (β = −0.091, *p* = 0.010), and advanced cancer stage (β = 1.932, *p* = 0.008). **Conclusions:** Tunisian cancer survivors experience substantial, multidimensional barriers to physical activity, with inadequate healthcare guidance representing a critical system-level gap. Findings support the development of culturally adapted, multidisciplinary interventions that target modifiable cognitive and symptom-related barriers, while enhancing patient activation and healthcare provider engagement.

## 1. Introduction

Cancer survivorship represents a rapidly expanding global health priority. The population of patients living with a cancer diagnosis reached 18.1 million individuals in the United States in 2022, with projections of 26 million by 2040, reflecting improved survival rates due to advances in detection and treatment strategies [1,2]. This demographic expansion necessitates comprehensive survivorship care that addresses long-term health outcomes and optimizes quality of life.

Physical activity emerges as a cornerstone intervention for cancer survivors, with meta-analyses demonstrating associations with 9–15% reductions in all-cause mortality and 9–27% reductions in cancer-specific mortality across multiple cancer types [3,4]. Beyond survival outcomes, regular physical activity improves cardiorespiratory fitness, muscle strength, and bone health, and enhances psychological well-being, while reducing treatment-related side effects, including fatigue, anxiety, and depression [5,6].

Tunisia confronts a mounting cancer burden, with age-standardized incidence increasing to 120.9 per 100,000 in 2020, alongside an estimated 51,348 prevalent cases over five years [7]. Lung cancer predominates in males (21.8% of cases) while breast cancer leads in females (35.4%), reflecting patterns observed in transitioning economies [8]. Cancer survivorship care in Tunisia remains underdeveloped, characterized by surveillance-focused, oncologist-centric approaches contrasting with integrated, multidisciplinary models adopted in high-resource settings [9,10].

Physical inactivity contributes significantly to cancer-related morbidity and mortality in Tunisia. Recent epidemiological data demonstrate that insufficient physical activity was responsible for 922 cancer-related deaths (9.8% of total cancer mortality) in 2019, including 431 lung cancer deaths (population attributable fraction: 17.2%), 141 colorectal cancer deaths (12.3%), and 101 post-menopausal breast cancer deaths (12.9%) [11]. Additionally, 22,855 disability-adjusted life years (9.9% of total cancer-related DALYs) were attributed to insufficient physical activity, underscoring the substantial preventive potential of activity interventions [11].

Previous investigations of exercise barriers and facilitators in cancer survivors have predominantly focused on Western populations [12,13]. Cultural factors, socioeconomic conditions, healthcare infrastructure, family support systems, and religious considerations may differ substantially between Western and Middle East and North Africa (MENA) contexts, potentially limiting the applicability of existing findings [14,15]. This cultural specificity is particularly relevant given that health behaviours are deeply embedded within cultural frameworks and social determinants of health.

Validated instruments enable systematic assessment of physical activity barriers among cancer survivors. The Physical Activity Barriers After Cancer (PABAC) scale represents a 12-item tool specifically developed to identify challenges following cancer diagnosis and treatment, covering four domains: symptoms (fatigue, pain, nausea, depression), cognitive (motivation, discipline, time constraints), logistical (financial resources, safe environment), and clinical (surgical complications, medical contraindications) [16,17]. The Patient Activation Measure (PAM-13) assesses individuals’ knowledge, skills, and confidence in health self-management and demonstrates strong associations with health behaviors in populations with chronic diseases [18,19].

Several critical gaps exist in cancer survivorship research within the MENA region. First, limited data exist regarding physical activity patterns and associated barriers among Tunisian cancer survivors, representing an unaddressed population-specific knowledge deficit. Second, the relationship between patient activation and physical activity engagement remains unexplored in MENA cancer survivors, despite demonstrated importance in Western populations. Third, healthcare system factors influencing exercise guidance and support have not been systematically evaluated in Tunisian oncology settings. Fourth, cultural and socioeconomic determinants of physical activity behavior specific to the Tunisian context require investigation to inform culturally appropriate interventions.

Our study aimed to: (i) comprehensively assess physical activity patterns and perceived barriers among Tunisian cancer survivors post-chemotherapy; (ii) examine relationships between patient activation, fatigue severity, and physical activity engagement; (iii) evaluate the current healthcare system provision of exercise guidance and support; and (iv) determine the factors associated with higher perceived physical activity barriers, thereby providing descriptive data for future intervention design.

We hypothesized that: (i) Tunisian cancer survivors would demonstrate substantial barriers to physical activity, with symptom-related and cognitive factors predominating; (ii) patient activation levels would correlate inversely with barrier perceptions and positively with activity engagement; (iii) healthcare guidance provision would be limited; and (iv) fatigue severity and advanced cancer stage would independently predict barrier perceptions.

## 2. Methods

### 2.1. Ethical Approval

The study protocol received approval from the Ethical Committee of the Faculty of Medicine Ibn El Jazzar, University of Sousse, Tunisia (reference CEFMSo_0065_2025) on 24 April 2025. All participants provided informed consent following study explanation and confidentiality assurance. The study complied with the principles of the Declaration of Helsinki and the Good Clinical Practice guidelines.

### 2.2. Study Design and Setting

This cross-sectional analytical study was conducted through collaboration between the Physical Medicine and Rehabilitation Department at Sahloul Hospital and the Oncology Department at University Hospital Farhat Hached, Sousse, Tunisia. Data collection was conducted via structured telephone interviews following medical record screening to determine eligibility. To mitigate response bias (e.g., social desirability and respondent fatigue), two key procedural controls were implemented. We first ensured data reliability by emphasizing confidentiality and anonymity in the consent process. Subsequently, interview duration was rigorously monitored (average 25 min) to minimize respondent fatigue.

### 2.3. Sample Size Calculation

Sample size was calculated using G*Power 3.1.9.7, assuming a medium effect size (f^2^ = 0.15) for multivariable regression analysis with eight predictors, α = 0.05, power = 0.80, yielding *n* = 109. Accounting for a 10% non-response rate, the target sample size was 120 participants.

### 2.4. Participants and Eligibility Criteria

Participants were recruited from oncological follow-up care registries at University Hospital Farhat Hached using systematic sampling. Inclusion criteria: (a) age ≥ 18 years; (b) confirmed cancer diagnosis with completed chemotherapy ≥ 3 months prior; (c) any cancer type eligible; (d) hormone therapy recipients included; (e) completed indicated radiotherapy; (f) informed consent provided. Exclusion criteria: (a) current chemotherapy; (b) incomplete chemotherapy; (c) pending radiotherapy; (d) immunotherapy/targeted therapy recipients (to ensure homogeneous chemotherapy-exposed sample); (e) severe pre-diagnosis comorbidities limiting physical activity; (f) cognitive/psychiatric impairment preventing interview completion; (g) discontinued care; (h) missing contact information.

### 2.5. Outcome Measures

#### 2.5.1. Demographic and Clinical Variables

They included age, gender, anthropometric measures, education, employment, insurance status, cancer characteristics, treatment history, time since diagnosis/chemotherapy, and comorbidities.

#### 2.5.2. Physical Activity Assessment

IPAQ-SF assessed activity levels during the previous seven days using the validated Arabic version [20,21]. Both categorical (inactive, minimally active, health-enhancing physical activity, and active) and continuous (MET minutes/week) scores were calculated according to established protocols. Pre-chemotherapy and current activity levels were assessed through structured recall questionnaires.

#### 2.5.3. Barriers Assessment

PABAC scale evaluated 12 obstacles using 4-point Likert scales (1 = strongly disagree, 4 = strongly agree) [16,17]. Four subscales assess symptoms, cognitive barriers, logistical barriers, and clinical barriers. Total scores range from 12 to 48, with higher scores indicating greater barriers.

#### 2.5.4. Fatigue Assessment

FAS measured severity using 10 items on 5-point scales [22]. The validated Arabic version assessed physical and mental fatigue dimensions using the following categories: no fatigue (<22), moderate (22–34), severe (≥35) [23].

#### 2.5.5. Patient Activation

PAM-13 evaluated health self-management knowledge, skills, and confidence [18,19]. Validated Arabic version generated interval-level scores (0–100) with four activation levels: Level 1 (0–47, disengaged), Level 2 (47.1–55.1, becoming aware), Level 3 (55.2–72.4, taking action), Level 4 (72.5–100, maintaining behaviors).

#### 2.5.6. Exercise Guidance and Facilitators

Structured questions assessed healthcare professional guidance, information sources, motivation levels, and program interest.

### 2.6. Statistical Analysis

Analyses were performed using IBM SPSS version 29.0. Normality was assessed using Shapiro–Wilk tests and visual inspection. Descriptive statistics included frequencies/percentages for categorical variables, means ± standard deviations for normally distributed continuous variables, and medians with interquartile ranges (IQRs) for non-normally distributed continuous variables.

ROC curve analysis evaluated PABAC’s performance in predicting physical inactivity, calculating the area under the curve (AUC), sensitivity, and specificity, with 95% confidence intervals. Optimal cut-off was determined using Youden’s index.

Pre- and post-chemotherapy comparisons used McNemar’s test for categorical variables and the Wilcoxon signed-rank test for continuous variables. Effect sizes were calculated using Cohen’s conventions.

Correlation analyses employed Pearson (normal distributions) or Spearman (non-normal) coefficients with strength categorization: very strong (≥0.70), strong (0.50–0.69), moderate (0.30–0.49), weak (<0.30). Between-group comparisons used Student’s *t*-tests or Mann–Whitney U tests for continuous variables, and chi-square tests for categorical variables.

Multivariable linear regression models identified independent factors associated with PABAC and PAM scores, including variables with *p* < 0.10 in univariable analyses to minimize Type II error. Model assumptions were verified through residual analysis. Bonferroni correction was applied for multiple comparisons. Statistical significance was set at *p* ≤ 0.05 with 95% confidence intervals reported, unless otherwise specified.

## 3. Results

### 3.1. Participant Characteristics

Complete data were available for 119 participants (99.2% response rate). Mean age was 51.89 ± 10.2 years (range: 19–74), with predominant representation in the fourth (43.7%) and fifth (27.7%) decades. Female participants represented 73.9% (*n* = 88) of the sample. Most were married (68.9%) and unemployed (58.8%), with 29.5% reporting illiteracy and 23.5% having a university education. Social insurance coverage was present in 70.6% of cases.

Table 1 presents comprehensive sociodemographic and clinical characteristics.

Mean BMI was 26.44 ± 3.74 kg/m^2^, with 68.1% classified as overweight/obese. Comorbidities affected 40.3%, predominantly hypertension (31.9%). Breast cancer represented the most common diagnosis (68.1%), followed by lung (14.3%) and colorectal (10.1%) cancers. Advanced-stage disease (Stage III–IV) was present in 59.7%. Surgery was performed in 92.4%, with 23.6% reporting persistent surgical pain. Radiotherapy was received by 89.9%. Median time since diagnosis was 29 months (IQR: 23–40); median time since chemotherapy completion was 14 months (IQR: 10–20).

### 3.2. Physical Activity Patterns

Substantial activity declines occurred post-chemotherapy. Table 2 outlines the various parameters assessed using IPAQ.

Vigorous activity participation decreased from 4.2% to 0% (*p* = 0.125, Fisher’s exact test), moderate activity from 31.1% to 1.7% (*p* < 0.001, McNemar’s test), and walking from 76.5% to 52.9% (*p* < 0.001). Total activity participation declined from 76.5% to 52.9% (*p* < 0.001). Median activity intensity decreased significantly from 297 MET-min/week (IQR: 99–517) to 44 MET-min/week (IQR: 0–198; *p* < 0.001, Wilcoxon test), representing a large effect size (r = 0.65). This decline affected 84 participants (70.6%).

IPAQ categorization revealed 47.1% inactive, 35.3% minimally active, and 17.6% at health-enhancing levels of physical activity. Sedentary time increased from 3 h/day (IQR: 2–3) to 5 h/day (IQR: 5–6; *p* < 0.001).

### 3.3. Barriers to Physical Activity

Mean total PABAC score was 29.72 ± 5.13 (range: 19–39), indicating substantial barriers. Cognitive barriers demonstrated the highest subscale scores (2.85 ± 0.58), followed by logistical (2.63 ± 1.03), symptoms (2.62 ± 0.57), and clinical barriers (1.39 ± 0.52).

Table 3 illustrates the intensity levels of various barriers to physical activity, as assessed by the PABAC scale.

The scores of the four sub-dimensions were calculated through averaging the ratings assigned to the corresponding items. Table 4 shows the physical activity barriers score.

Fatigue was nearly universal (80.7% strongly agreeing), followed by difficulty remaining disciplined (42%) and getting motivated (42%). Pain affected substantial proportions (23.5% strongly agreeing), while sadness/depression was prominent (45.4%). Financial limitations affected 40.3%, and environmental safety concerns affected 16%.

PABAC demonstrated excellent predictive performance for physical inactivity (AUC = 0.805, 95%CI: 0.724–0.887, *p* < 0.001). Optimal cut-off of ≥31 yielded sensitivity of 80.4%, specificity of 71.4%, positive predictive value of 79.2%, and negative predictive value of 72.7%.

### 3.4. Fatigue Assessment

Mean FAS score was 31.03 ± 4.02 (range: 22–42), with 100% reporting fatigue symptoms. Moderate fatigue (FAS 22–34) affected 79%, severe fatigue (FAS ≥ 35) affected 21%. No participants demonstrated the absence of fatigue.

### 3.5. Patient Activation

Mean PAM score was 49.84 ± 14.76, corresponding to Level 2 activation. Distribution: Level 1 (38.7%), Level 2 (30.3%), Level 3 (21%), Level 4 (10.1%). Only 31.1% achieved adequate activation (Levels 3–4).

Table 5 presents the distribution of patients by level of engagement in physical activities, as assessed by the PAM scale.

### 3.6. Exercise Guidance and Facilitators

Healthcare guidance was minimal: 26.1% received any information, with sources including healthcare professionals (35.5%), family/friends (35.5%), social media (19.4%), and other patients (9.6%). Among those receiving guidance from a healthcare professional (*n* = 11), only 45.5% felt motivated, and merely 4.2% of the total sample received adequate information.

Table 6 summarizes the frequency and characteristics of guidance on physical activity practice.

Despite limited guidance, 91.6% expressed interest in personalized exercise plans, 89.9% indicated willingness to participate in structured programs, 70.6% found group sessions appealing, and 64.7% considered online resources helpful. No participants had enrolled in cancer-specific programs.

Primary motivational factors included preventing recurrence (84.9%), remaining healthy/productive (74.8%), improving sleep/mental health, and increasing confidence.

### 3.7. Correlation Analyses

PABAC demonstrated very strong positive correlation with FAS scores (r = 0.704, *p* < 0.001), strong negative correlations with PAM scores (r = −0.679, *p* < 0.001) and activity intensity (r = −0.597, *p* < 0.001), moderate positive correlation with sedentary time (r = 0.385, *p* < 0.001), and weak positive correlation with age (r = 0.186, *p* = 0.043).

Table 7 presents correlations of the PABAC-12 score with quantitative variables.

PAM demonstrated strong positive correlation with activity intensity (r = 0.665, *p* < 0.001), strong negative correlation with PABAC (r = −0.679, *p* < 0.001), FAS scores (r = −0.677, *p* < 0.001), and sedentary time (r = −0.518, *p* < 0.001), moderate negative correlation with age (r = −0.389, *p* < 0.001).

Table 8 shows correlations between the PAM-13 score and quantitative variables.

### 3.8. Group Comparisons

PABAC scores were significantly higher among participants with advanced cancer stage (31.89 ± 4.34 vs. 26.52 ± 4.52, *p* < 0.001, Cohen’s d = 1.21), lower education (32.09 ± 3.88 vs. 26.87 ± 5.02, *p* < 0.001, d = 1.15), inadequate insurance (32.37 ± 2.84 vs. 28.62 ± 5.47, *p* < 0.001, d = 0.85), unemployment (30.87 ± 4.89 vs. 28.08 ± 5.07, *p* = 0.003, d = 0.57), and absence of exercise guidance (30.67 ± 4.73 vs. 27.03 ± 5.33, *p* = 0.001, d = 0.72).

PAM scores were significantly lower among participants with advanced cancer stage (44.18 ± 12.51 vs. 58.21 ± 13.94, *p* < 0.001, d = 1.07), lower education (42.26 ± 12.92 vs. 58.97 ± 11.34, *p* < 0.001, d = 1.36), inadequate insurance (42.71 ± 10.42 vs. 52.81 ± 15.33, *p* = 0.001, d = 0.78), unemployment (45.00 ± 15.03 vs. 56.76 ± 11.31, *p* < 0.001, d = 0.88), and absence of exercise guidance (46.08 ± 13.32 vs. 60.51 ± 13.57, *p* < 0.001, d = 1.07).

### 3.9. Multivariable Analysis

Independent factors associated with higher barriers (PABAC) included fatigue severity (β = 0.466, 95%CI: 0.250–0.683, *p* < 0.001), lower patient activation (β = −0.091, 95%CI: −0.159 to −0.022, *p* = 0.010), and advanced cancer stage (β = 1.932, 95%CI: 0.508–3.356, *p* = 0.008). The model explained 57.3% of variance (R^2^ = 0.573, F = 49.6, *p* < 0.001).

Independent factors associated with higher activation (PAM) included younger age (β = −0.251, 95%CI: −0.421 to −0.081, *p* = 0.004), employment (β = 4.263, 95%CI: 0.749–7.777, *p* = 0.018), higher activity intensity (β = 0.014, 95%CI: 0.002–0.025, *p* = 0.020), lower barrier scores (β = −0.662, 95%CI: −1.161 to −0.163, *p* = 0.010), and lower fatigue scores (β = −0.794, 95%CI: −1.408 to −0.180, *p* = 0.012). The model explained 63.1% of variance (R^2^ = 0.631, F = 24.3, *p* < 0.001).

Table 9 presents independent factors associated with high PABAC and PAM scores.

## 4. Discussion

This investigation provides the first comprehensive assessment of physical activity barriers and facilitators among Tunisian cancer survivors, revealing substantial obstacles alongside significant opportunities for intervention. The findings demonstrate marked declines in activity post-chemotherapy, with cognitive and symptom-related barriers predominating over logistical factors, while patient activation levels remained suboptimal and healthcare guidance was minimal.

### 4.1. Physical Activity Decline: Magnitude and Interpretation

The 85% reduction in activity intensity (297 to 44 MET-min/week) substantially exceeds declines reported in most Western populations, where reductions typically range 30–60% [24,25]. This divergence likely reflects multiple contextual factors. Unlike Western healthcare systems, which have established survivorship care pathways and rehabilitation services, Tunisia’s oncologist-centric model provides limited post-treatment support [9,10]. Although physical inactivity represents a persistent global health challenge, our findings underscore regional-specificities in the MENA context, where cultural norms during illness recovery often prioritize rest and family support over active rehabilitation [14,15].

The complete absence of vigorous activity post-chemotherapy, in contrast to maintenance of some vigorous activity in Western cohorts [24,25], suggests particularly severe functional decline. This pattern likely reflects inadequate pre-treatment fitness assessments, the absence of rehabilitation programs, and a lack of graduated return-to-activity protocols. The magnitude of decrease places Tunisian cancer survivors at elevated risk for cardiovascular complications, metabolic dysfunction, and potentially cancer recurrence [26,27].

These findings align with broader patterns observed in transitioning economies, where cancer care infrastructure tends to focus on acute treatment delivery rather than comprehensive survivorship support [28]. The increased sedentary time (3 to 5 h daily) compounds the effects of reduced activity, creating a deconditioning spiral that may be particularly difficult to reverse without structured interventions.

### 4.2. Barrier Patterns: Cultural and System-Level Determinants

Cognitive barriers predominate (mean 2.85 ± 0.58), contrasting with Western studies, where symptom-related barriers often dominate [12,13]. This divergence likely reflects cultural factors influencing health behaviour, self-efficacy, and lack of control. Traditional Middle Eastern healthcare relationships prioritize provider authority and patient compliance over collaborative decision-making and self-management [29,30]. Consequently, survivors may lack confidence in making independent decisions about activities, particularly following a serious illness.

The high endorsement of motivation difficulties (42%) and discipline challenges (42%) suggests barriers beyond symptom burden. This pattern may reflect cultural beliefs about cancer as a punishment or test requiring passive acceptance rather than active engagement [31]. Religious frameworks that emphasize submission to divine will, while providing psychological comfort, may inadvertently discourage proactive health behaviors [32].

Fatigue universality (100% affected) exceeds rates reported in comparable Western studies (70–90%) [32,33]. This divergence may reflect measurement differences, but it is more likely to indicate genuine population differences in fatigue experience. Potential explanations include: (1) later-stage diagnosis patterns in resource-limited settings leading to more intensive treatments; (2) limited supportive care during treatment resulting in greater residual toxicity; (3) cultural differences in fatigue expression and reporting; and (4) absence of evidence-based fatigue management interventions.

The very strong correlation between fatigue and barriers (r = 0.704) confirms fatigue as the primary modifiable target for intervention. However, effective fatigue management requires systematic assessment, evidence-based treatments, and longitudinal monitoring. These resources are currently absent from Tunisian cancer care [34,35].

### 4.3. Patient Activation: A Critical Point of Intervention

The suboptimal PAM score (49.84) indicates systematic deficiencies in patient empowerment. Patient activation refers to an individual’s knowledge, skills, and confidence in managing their own health [18,19]. The strong correlations between PAM scores and both barrier perceptions (r = −0.679) and activity levels (r = 0.665) demonstrate the central role of activation in determining health behavior.

This finding aligns with Western research demonstrating activation as a modifiable predictor of health outcomes [36,37]. However, the magnitude of the correlation exceeds that reported in most studies, suggesting that activation may be particularly influential in contexts where healthcare systems provide limited ongoing support. When providers offer minimal guidance, patients must rely heavily on internal resources. This makes activation even more critical for positive outcomes.

The predominance of Level 1 and 2 activation (69%) indicates that most survivors lack confidence and skills for effective self-management of their health. This pattern likely reflects traditional paternalistic healthcare relationships where patients receive little education about self-care and are discouraged from questioning medical authority [29,30]. A transformation toward collaborative care models that emphasize patient empowerment is a fundamental requirement for sustainable behavior change.

### 4.4. Healthcare System Gaps: Critical Areas for Reform

The minimal healthcare guidance (26.1% receiving any information, 4.2% receiving adequate guidance) represents a critical system failure requiring immediate attention. This contrasts sharply with evidence-based recommendations that emphasize provider counseling as essential for activity promotion [38,39]. The finding that only 45.5% of those receiving professional guidance felt motivated suggests that current counselling approaches are deficient in quality.

Several factors likely contribute to this gap: (1) provider’s time constraints in resource-limited settings; (2) lack of training in behaviour change counselling; (3) absence of standardized guidelines for exercise prescription in cancer survivors; (4) limited awareness of physical activity benefits among oncology providers; and (5) absence of allied health professionals trained in cancer exercise programming.

The high interest in structured programs (89.9%), group sessions (70.6%), and online resources (64.7%) indicates substantial unmet demand for comprehensive support. This demand-supply mismatch represents both a challenge and an opportunity for the development of the healthcare system. Unlike resource-intensive medical interventions, many physical activity programs can be delivered cost-effectively through community partnerships and digital platforms.

### 4.5. Clinical and Policy Implications

These findings necessitate comprehensive, multi-level interventions that address individual, healthcare system, and policy factors. At the personal level, evidence-based approaches should target the primary modifiable barriers identified, including fatigue management through graduated exercise programs combined with cognitive-behavioral strategies to enhance motivation and self-efficacy [40,41]. Improving patient activation through structured education, shared decision-making training, and development of self-monitoring skills represents a high-leverage intervention with demonstrated effectiveness [42].

Healthcare system transformation requires provider training in behaviour change counselling, development of clinical practice guidelines for exercise prescription, and integration of allied health professionals into survivorship care teams [5,43]. Establishing cancer rehabilitation services based on successful international models could address the comprehensive needs of survivors while providing specialized expertise in exercise programming [44,45].

Policy interventions should address structural determinants through employment protection legislation, insurance coverage for rehabilitation services, and community infrastructure development that supports access to physical activity [46,47]. Media campaigns highlighting survivor stories and the benefits of exercise could begin to shift cultural attitudes toward more proactive recovery approaches.

### 4.6. Study Limitations

Several limitations warrant consideration. Cross-sectional designs prevent causal inference regarding barrier-behavior relationships, particularly when measuring direct cultural factors. The predominantly female breast cancer sample may limit generalizability, though this composition reflects regional epidemiological patterns and healthcare utilization behaviours. Selection bias introduced by telephone recruitment may have excluded participants without reliable access to communication. This potentially underestimates the barriers faced by the most vulnerable populations. Healthy Survivor Bias must be considered, as the exclusion of patients who may have died shortly after chemotherapy likely leads to an overestimation of characteristics within the healthiest patient subset.

The absence of objective physical activity measurement through accelerometry represents a methodological limitation, though validated self-report instruments provide acceptable alternatives for large-scale epidemiological studies [48]. Cultural adaptation of measurement tools, even when using established translated versions, may not capture all relevant cultural concepts related to physical activity and health behaviors.

### 4.7. Future Research Directions

Future research priorities include longitudinal studies that track the evolution of barriers and the response to interventions over extended follow-up periods. Randomized controlled trials testing culturally adapted interventions targeting identified barriers are essential to developing evidence-based practice. Mixed-methods research could provide a deeper understanding of the cultural factors and family dynamics that influence health behaviors.

Implementation research examining optimal service delivery models, provider training approaches, and policy interventions would support a systematic transformation of the healthcare system. Economic evaluations would inform resource allocation decisions and demonstrate the value of interventions to healthcare administrators and policymakers.

### 4.8. Educational Priorities

The minimal healthcare guidance provision (4.2% receiving adequate information) mandates urgent reforms to the education system. Tunisian universities must establish specialized master’s programs in clinical exercise physiology with dedicated oncology tracks to train qualified professionals capable of delivering evidence-based exercise interventions. These programs should emphasize cultural competency, Arabic language proficiency, and an understanding of Islamic health perspectives to ensure culturally responsive care delivery.

Continuing medical education initiatives targeting oncologists, nurses, and allied health professionals are essential. Professional development curricula should include principles of exercise prescription, motivational interviewing techniques, and patient activation strategies. The Tunisian Ministry of Health should mandate annual survivorship care training requirements for oncology providers, with certification maintenance dependent on demonstrated competency in exercise counselling.

Patient education transformation requires developing culturally adapted materials addressing the cognitive barriers identified (motivation, discipline, self-efficacy). Interactive educational platforms in Arabic and French, featuring local survivor testimonials and endorsements from religious leaders, could help address cultural attitudes toward physical activity during recovery. Community health worker programs should be expanded to include survivorship care specialists who can provide ongoing education and support in patients’ homes and communities.

### 4.9. Healthcare System Integration

Clinical exercise physiologists must be integrated into standard cancer care teams at major Tunisian hospitals. These specialists should provide individualized exercise prescriptions, fatigue management strategies, and ongoing monitoring throughout the survivorship continuum. The current study’s findings, demonstrating a strong correlation between patient activation and activity engagement (r = 0.665), support embedding activation-enhancing interventions into routine clinical workflows.

Cancer rehabilitation centers should be established based on successful international models, offering comprehensive services including physical therapy, exercise programming, nutritional counseling, and psychological support. These centers should emphasize the peer support programs that 70.6% of participants found appealing, creating community networks that extend beyond formal healthcare interactions.

Telemedicine platforms specifically designed for cancer survivors could address geographical barriers while providing ongoing support. These platforms should include remote monitoring, virtual group exercise sessions, and direct communication with clinical exercise physiologists. Given the high interest in online resources (64.7%), digital health initiatives represent particularly promising intervention modalities.

### 4.10. Policy and Insurance Reform

Expanding insurance coverage to include survivorship care services is essential to the program’s sustainable implementation. The current study’s demonstration that inadequate insurance is associated with greater barriers (effect size, d = 0.85) highlights insurance reform as a critical point of intervention. The National Health Insurance Fund (CNAM) should develop specific reimbursement categories for cancer rehabilitation services, including exercise physiology consultations, structured exercise programs, and interventions for managing fatigue.

Workplace protection policies for cancer survivors require strengthening, given the strong associations between employment status and both barriers and activation. National legislation should mandate employer accommodation for cancer survivors, including flexible scheduling for medical appointments, graduated return-to-work programs, and protection against employment discrimination. These policies should be coupled with employer education initiatives that highlight the business benefits of supporting survivors.

Public infrastructure development should prioritize creating safe, accessible exercise environments specifically designed for vulnerable populations. Community centers, walking paths, and outdoor exercise equipment should be strategically located in areas with high concentrations of cancer survivors, with programming specifically targeting the unique needs of this population.

### 4.11. Community and Cultural Interventions

Faith-based organization partnerships represent untapped resources for intervention delivery, given the central role of religious institutions in Tunisian communities. Islamic leaders should be engaged in developing theologically informed messages about physical activity as a form of self-care and stewardship, countering cultural beliefs that may discourage proactive health behaviors.

Survivor peer support networks should be formalized and funded, building on the high interest in group programs. Trained survivor volunteers could provide mentorship, accompany newly diagnosed patients to exercise programs, and serve as living examples of successful recovery and health maintenance.

Media campaigns featuring local survivor stories should challenge cultural narratives emphasizing rest and passivity during cancer recovery. These campaigns should highlight Islamic principles of health preservation, family responsibilities that require maintaining physical function, and community benefits of survivor engagement.

### 4.12. Research and Implementation Priorities

Longitudinal implementation studies should track the effectiveness of multi-level interventions, with particular attention to cultural adaptation requirements and sustainability factors. Economic evaluations demonstrating the cost-effectiveness of survivorship programs will be crucial for securing ongoing support from governments and institutions.

Regional collaboration initiatives should utilize shared cultural and linguistic similarities across MENA countries to develop scalable intervention models. International partnerships with established cancer rehabilitation programs could accelerate knowledge transfer while ensuring cultural appropriateness.

Training infrastructure development requires partnerships between Tunisian institutions and international universities with established clinical exercise physiology programs. Faculty exchange programs, curriculum sharing agreements, and joint research initiatives could rapidly build local expertise while maintaining global quality standards.

### 4.13. Implementation Roadmap

Based on our findings and experience, we propose the following roadmap for the development and expansion of cancer rehabilitation services in Tunisia. During Phase 1 (Years 1–2), efforts should focus on establishing clinical exercise physiology training programs, initiating provider education curricula, and piloting cancer rehabilitation services at major hospitals. In Phase 2 (Years 3–4), the aim will be to scale up the most successful pilot programs, implement insurance coverage reforms to ensure sustainability, and launch community-based interventions in collaboration with faith-based organizations. Finally, in Phase 3 (Years 5 and beyond), the goal is to achieve nationwide coverage of survivorship care services, position Tunisia as a regional model for cancer rehabilitation, and initiate international collaboration programs to strengthen capacity building across the MENA region.

### 4.14. Regional and Global Implications

This investigation provides foundational evidence that systematic transformation of cancer survivorship care is both necessary and feasible within resource-constrained healthcare systems. The substantial interest in intervention (91.6% wanting personalized programs) indicates significant potential for successful implementation, provided that interventions address the cultural, educational, and systemic barriers identified. This necessity is formally recognized at the institutional level, as demonstrated by recent efforts, including the 2023 adaptations of the National Comprehensive Cancer Network (NCCN) Guidelines for the MENA region [49]. These regional modifications acknowledge that effective clinical direction requires factoring in local resource availability, practice patterns, and cultural dynamics, thereby validating our study’s central premise that locally derived data are essential for appropriate survivorship care.

The integration of clinical exercise physiologists into cancer care teams represents a paradigm shift from traditional biomedical approaches toward comprehensive, patient-centered survivorship models. This transformation could position Tunisia as a regional leader in cancer rehabilitation while providing a replicable model for similar healthcare systems throughout the developing world.

The findings demonstrate that patient activation serves as a critical point of intervention for health behavior change, particularly in contexts where healthcare system support is limited. Investment in patient empowerment strategies may yield disproportionate returns through enabling survivors to navigate barriers independently while advocating for their own care needs.

Ultimately, this research reveals that the barriers facing Tunisian cancer survivors are not insurmountable obstacles, but systematic challenges requiring coordinated, evidence-based responses. The comprehensive intervention framework proposed provides a roadmap for transforming survivorship care delivery that could serve as a model for healthcare systems throughout the MENA region and other resource-limited settings globally.

## 5. Conclusions

This investigation reveals that Tunisian cancer survivors face profound, multidimensional barriers to physical activity engagement, with systematic healthcare delivery gaps requiring immediate, comprehensive transformation. Survivors face both physical and psychological obstacles, compounded by weak healthcare support. The findings provide a blueprint for evidence-based interventions at the educational, clinical, policy, and community levels that could transform cancer survivorship care across the MENA region.

## Figures and Tables

**Table 1:** Sociodemographic and Clinical Data.

Parameter	(N, %, Mean ± SD)
**Mean age (years)**	51.89 ± 10.2
**Gender**	
Male	31, 26.1%
Female	88, 73.9%
**Marital status**	
Married	82, 68.9%
Single	13, 10.9%
Divorced	11, 9.3%
Widowed	13, 10.9%
**Education level**	
Illiterate	35, 29.5%
Primary	30, 25.2%
Secondary	26, 21.8%
University	28, 23.5%
**Employment status**	
Not working	70, 58.8%
Working (blue collar)	25, 21%
Working (white collar)	24, 20.2%
**Social security**	
Type I	24, 20.2%
Type II	11, 9.2%
CNAM	84, 70.6%
**Body mass index (kg/m^2^): mean ± SD**	26.44 ± 3.74
Normal	38, 31.9%
Overweight	67, 56.3%
Obesity	14, 11.8%
**Co-morbidities**	48, 40.3%
HTA	38, 31.9%
Diabetes	16, 13.4%
Dyslipidaemia	9, 7.6%
Arthrosis	5, 4.2%
Hypothyroidism	4, 3.4%
Adrenal insufficiency	1, 0.8%
Guillain-Barré syndrome sequelae	1, 0.8%
Osteoporosis	5, 4.2%
**Evolution**	
Time since diagnosis (months): median	29 [23–40]
Time since last chemotherapy (months): median	14 [10–20]

(The parameter title is highlighted **in bold**).

**Table 2:** Distribution of Patients by Intensity and Duration Corresponding to Each Type of Physical Activity (IPAQ).

	Before Chemotherapy		After Chemotherapy	
	*n* (%)	Intensity/Duration	*n* (%)	Intensity/Duration
Vigorous Activity *	5 (4.2%)	960 [480–1560]	0 (0%)	-
Moderate Activity *	37 (31.1%)	240 [220–480]	2 (1.7%)	720
Walking *	91 (76.5%)	297 [198–396]	63 (52.9%)	198 [99–264]
Sedentary time **	-	3 [2–3]	-	5 [5–6]
Total physical activity	91 (76.5%)	396 [198–594]	63 (52.9%)	198 [99–297]

* Intensity (MET-min/week); ** Duration (h/day).

**Table 3:** Profile of Patients According to Physical Activity Barriers Assessed by the PABAC Scale.

		Strongly Disagree	Disagree	Agree	Strongly Agree
Cognitive	(N, %)				
	Not enough time/too busy	39, 32.8%	54, 45.4%	15, 12.6%	11, 9.2%
	Difficulty getting motivated	3, 2.5%	11, 9.2%	55, 46.2%	50, 42.0%
	Difficulty remaining disciplined	2, 1.7%	10, 8.4%	57, 47.9%	50, 42.0%
Logistical	(N, %)				
	Lack of a safe environment to exercise	27, 22.7%	28, 23.5%	45, 37.8%	19, 16.0%
	Lack of financial resources to exercise	27, 22.7%	17, 14.3%	27, 22.7%	48, 40.3%
Symptoms	(N, %)				
	Nausea	108, 90.8%	7, 5.9%	3, 2.5%	1, 0.8%
	Fatigue	1, 0.8%	2, 1.7%	20, 16.8%	96, 80.7%
	Pain	27, 22.7%	15, 12.6%	49, 41.2%	28, 23.5%
	Sadness/Depression	24, 20.2%	9, 7.6%	32, 26.9%	54, 45.4%
	Treatment side effects	32, 26.9%	19, 16.0%	37, 31.1%	31, 26.1%
Clinical	(N, %)				
	Surgical complications	71, 59.7%	23, 19.3%	20, 16.8%	5, 4.2%
	My doctor told me not to exercise	107, 89.9%	9, 7.6%	3, 2.5%	-

**Table 4:** Physical Activity Barriers Score (Subdimensions and Total PABAC Score).

	Mean ± SD	Min–Max
Cognitive scale	2.85 ± 0.58	1–4
Logistical scale	2.63 ± 1.03	1–4
Symptoms scale	2.62 ± 0.57	1.4–3.8
Clinical scale	1.39 ± 0.52	1–3
Total score PABAC	29.72 ± 5.13	19–39

**Table 5:** Distribution of Patients by their Level of Engagement in Physical Activities as Assessed by the PAM Scale.

	Number (*n*)	Frequency (%)
Level 1: Disengaged and overwhelmed	46	38.7
Level 2: Becoming aware but still struggling	36	30.3
Level 3: Taking action	25	21
Level 4: Maintaining behaviours and pushing further	12	10.1

**Table 6:** Frequency and Characteristics of Guidance on Physical Activity Practice.

	Number (*n*)	Frequency (%)
EXERCISE GUIDANCE		
Received guidance on exercise post-chemotherapy	31	26.1
Other patients	3	9.6
Family/Friends	11	35.5
Social media	6	19.4
Health care professionals (HCP)	11	35.5
Felt motivated by healthcare professionals’ advice *	5	45.5
HCP provided adequate information regarding safe physical activity	5	4.2
MOTIVATION		
Interested in receiving personalized exercise plans tailored to specific needs and limitations	109	91.6
Participation in any physical activity programs specifically designed for cancer patients	0	0
Group exercise sessions with other cancer survivors would be appealing	84	70.6
Online resources or virtual exercise classes would be helpful in promoting engagement in physical activity	77	64.7
Interested in participating in a structured exercise program designed for cancer patients	107	89.9

* *n* = 11.

**Table 7:** Correlation of the PABAC-12 Score with Quantitative Variables.

	*p*	r
Age	0.043 *	0.186
PAM score	<0.001 *	−0.679
FAS score	<0.001 *	0.704
Physical Activity post chemotherapy	<0.001 **	−0.597
Sitting time post chemotherapy	<0.001 **	0.385
Time since chemotherapy	0.098 **	-

* Pearson test; ** Spearman test.

**Table 8:** Correlation of the PAM-13 Score with Quantitative Variables.

	*p*	r
Age (years)	<0.001 *	−0.389
PABAC score	<0.001 *	−0.679
FAS score	<0.001 *	−0.677
Physical Activity post chemotherapy	<0.001 **	0.665
Sitting time post chemotherapy	<0.001 **	−0.518
Time since chemotherapy	0.227 **	-

* Pearson test; ** Spearman test.

**Table 9:** Independent Factors Associated with High PABAC and PAM Scores.

	PABAC Score		PAM Score	
	*p*	B [IC_95_%]	*p*	B [IC_95_%]
Age *	0.183	−0.044 [−0.109; 0.021]	0.004	−0.251 [−0.421; −0.081]
PAM score *	0.01	−0.091 [−0.159; −0.022]	-	-
PABAC score *	-	-	0.01	−0.662 [−1.161; −0.163]
FAS score *	<0.001	0.466 [0.25; 0.683]	0.012	−0.794 [−1.408; −0.18]
Physical Activity post chemotherapy *	0.068	−0.004 [−0.008; 0]	0.02	0.014 [0.002; 0.025]
Sitting time post chemotherapy *	0.882	0.046 [−0.565; 0.657]	0.192	−1.085 [−2.722; 0.552]
Tumor stage III–IV	0.008	1.932 [0.508; 3.356]	0.338	−1.921 [−5.878; 2.035]
Secondary-university level	0.136	−1.219 [−2.825; 0.388]	0.231	2.646 [−1.709; 7.002]
CNAM insurance	0.923	0.073 [−1.423; 1.569]	0.675	−0.857 [−4.895; 3.181]
Working	0.599	0.355 [−0.978; 1.689]	0.018	4.263 [0.749; 7.777]
No receiving guidance on exercise	0.426	−0.625 [−2.175; 0.924]	0.143	−3.097 [−7.253; 1.059]

* Risk calculated per one-unit increase.

## Data Availability

The datasets supporting the conclusions are available from the corresponding author upon reasonable request.
